# Clozapine prescribing during follow-up of a first-episode psychosis cohort

**DOI:** 10.1192/j.eurpsy.2021.2124

**Published:** 2021-08-13

**Authors:** R. Rowntree, F. Fanning, D. Keating, S. Murray, A. Szigeti, R. Doyle, S. Mcwilliams, M. Clarke

**Affiliations:** 1 Detect, St John of God Community Services, Dublin, Ireland; 2 Pharmacy, St John of God Hospitaller Services, Dublin, Ireland; 3 Psychiatry, St John of God Hospitaller Services, Dublin, Ireland

**Keywords:** first-episode psychosis, psychosis, treatment-resistant schizophrenia, clozapine

## Abstract

**Introduction:**

Of those with schizophrenia, one third develop treatment-resistant illness. Nearly 60% of these benefit from clozapine- the only antipsychotic medication licensed in this group.

**Objectives:**

As treatment-resistant illness developed in the follow-up of a first-episode psychosis (FEP) cohort, clozapine was prescribed. This study retrospectively compared the clozapine prescribing patterns, within this cohort, to National Institute for Health and Care Excellence (NICE) guidelines. In addition, impact on hospitalisation, physical health monitoring and augmentation strategies employed following clozapine initiation were examined. Factors delaying initiation of clozapine treatment or contributing to its discontinuation were also explored.

**Methods:**

The study included 339 individuals resident within an Irish community mental health team catchment area, referred with FEP from 1 January 2005 to 31 August 2016. Data were extracted from electronic medical records.

**Results:**

Within the cohort, clozapine was prescribed to 32 individuals (9.4%). The mean number of adequate trials of antipsychotic prior to starting clozapine was 2.74 (SD 1.13; range 1–5). The mean time to clozapine trial was 2.1 years (SD 1.95; range 0.17–6.25). Following initiation of clozapine, mean hospital admissions per year fell from 2.3 to 0.3 (p=0.00). Mean inpatient days pre- and post-clozapine also decreased (147 vs. 53; p=0.00). In all, 18 patients ceased use of clozapine, 5 temporarily and 13 permanently.

**Conclusions:**

Patients are being prescribed clozapine earlier than previously demonstrated. However, delayed treatment remains common, and many patients discontinue clozapine. Further research is necessary to describe and address factors which contribute to its discontinuation.

**Disclosure:**

No significant relationships.

